# Prior administration of a non-steroidal anti-androgen failed to prevent the flare-up caused by a luteinizing hormone-releasing hormone agonist in a patient with metastatic prostate cancer

**DOI:** 10.1186/s13104-015-1297-3

**Published:** 2015-08-05

**Authors:** Sho Uehara, Takeshi Yuasa, Yasuhisa Fujii, Akihiro Yano, Shinya Yamamoto, Hitoshi Masuda, Iwao Fukui, Junji Yonese

**Affiliations:** Department of Urology, Cancer Institute Hospital, Japanese Foundation for Cancer Research, 3-8-31 Ariake, Koto-ku, Tokyo, 135-8550 Japan

**Keywords:** Metastatic prostate cancer, Flare phenomenon, Anti-androgen withdrawal syndrome

## Abstract

**Background:**

‘Flare phenomenon’ after initial luteinizing hormone-releasing hormone agonist administration is a widely approved concept in the treatment of prostate cancer. In most guidelines, concomitant therapy with anti-androgens is recommended to prevent this flare phenomenon. However, there are few reports describing serum prostate-specific antigen transitions after hormonal therapy. Here, we present a case of a man who experienced the biochemical and clinical flare phenomenon despite prior anti-androgen use and who has detailed data.

**Case presentation:**

A 70-year-old Asian man with metastatic prostate cancer (multiple bone) was referred to our hospital. He was treated with prior anti-androgens and luteinizing hormone-releasing hormone agonist. Regardless of prior use of anti-androgens, his low back pain caused by bone metastases was deteriorated and serum prostate-specific antigen level was raised from 974.8 ng/mL to 2,555.5 ng/mL within 3 weeks. Then, his serum prostate specific antigen level started to decrease along with the pain. The nadir reached 1.0 ng/mL and remained for 6 months. Because the serum level of prostate-specific antigen then began to increase again, anti-androgen was discontinued for anti-androgen withdrawal syndrome. Then the serum level decreased again to less than 0.1 ng/mL. Until now, his serum prostate-specific antigen level has been maintained at less than 0.1 ng/mL for more than 30 months without any clinical progressions.

**Conclusion:**

We present the case of a patient in whom a clinical flare caused by an leuteinizing hormone-releasing hormone agonist was not prevented by prior anti-androgen administration. In addition, the nadir level of prostate-specific antigen when he received leuteinizing hormone-releasing hormone monotherapy was ten times lower than when he received concomitant therapy, and period of anti-androgen withdrawal syndrome was longer than usual. In this case, anti-androgen was probably not effective from the initial administration. Awareness of the possibility of ineffectiveness of anti-androgens is important in the treatment of symptomatic metastatic prostate cancer. Leuteinizing hormone-releasing hormone antagonist and surgical castration is a more reliable clinical approach for the prostate cancer patients with symptomatic metastatic disease.

## Background

Luteinizing hormone-releasing hormone (LH-RH) agonists are the ‘standard of care’ in hormonal therapy for the patients with advanced prostate cancer because they avoid the physical and psychological discomfort associated with orchiectomy [1]. However, potent initial detrimental effects, called the ‘flare phenomenon’, in advanced disease is a main concern at the initial LH-RH administration, and it includes increased bone pain, acute bladder outlet obstruction, obstructive renal failure and spinal cord compression [1–3]. The European Association of Urology guidelines recommend use of anti-androgens to prevent the clinical flare phenomenon [1]. However, in the clinical practice, the impact of the concomitant use of anti-androgen to avoid the clinical flare phenomenon, has practically been largely unknown. We report the case of a patient with metastatic prostate cancer who experienced the LH-RH agonist clinical flare despite preceding administration of non-steroidal anti-androgen.

## Case report

**Fig. 1:**
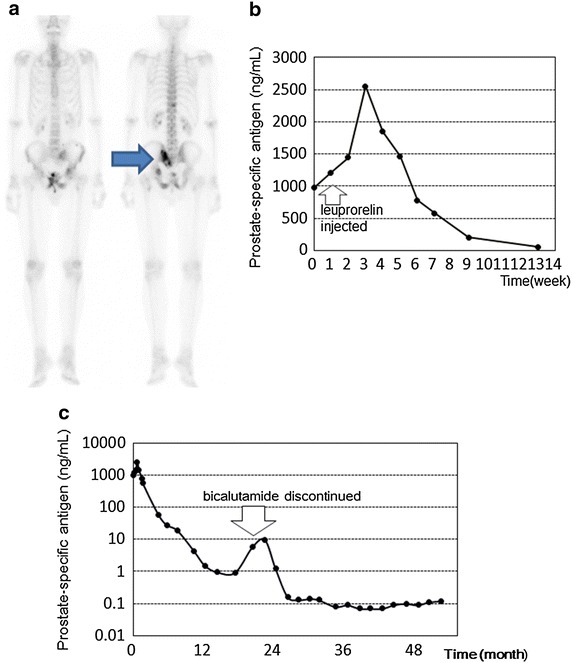
Imaging and the clinical schema of a symptomatic metastatic prostate cancer patient. **a** Initial bone scan images showing multiple bone metastases, which included the sacral bone (*arrow*). **b** Initial clinical schematic presentation with alteration of the serum prostate-specific antigen level. **c** Clinical schematic presentation of the whole process with alteration of the serum prostate-specific antigen level.

A 70-year-old Asian man was referred to our hospital for treatment of prostate cancer. His serum prostate-specific antigen (PSA) level was 974.8 ng/mL and trans-perineal prostatic biopsy revealed prostate cancer, with a Gleason score 5 + 4. A whole-body bone scintigraphy demonstrated multiple bone metastases [extent of disease (EOD): 2] including the sacral bone (Fig. 1a). Magnetic resonance imaging (MRI) also indicated a metastasis at the same site, which was causing severe low back pain. The patient was hospitalized for pain control and started to undergo hormonal therapy. Prior administration of the non-steroidal anti-androgen, bicalutamide, was followed by injection of the LHRH-agonist, leuprorelin, after 1 week. However, severe back pain continued to increase with increased PSA serum level (Fig. 1b). One week after bicalutamide administration, serum PSA level was elevated to 1,211.2 ng/mL, and to 1,443.8 ng/mL after 2 weeks. Three weeks after leuprorelin injection and 4 weeks after bicalutamide administration, while serum testosterone level had already been suppressed to castration levels (0.34 ng/mL), the serum PSA reached a peak of 2,555.5 ng/mL (Fig. 1b). Thereafter, the serum PSA level decreased along with the patient’s back pain. The serum testosterone was completely suppressed to a level of 0.1 ng/mL and PSA nadir reached 1.0 ng/mL and remained at approximately 1 ng/mL for 6 months (Fig. 1c). The PSA serum level then began to increase again without elevation of testosterone and any clinical symptoms, including back pain. For the purpose of anti-androgen withdrawal syndrome (AWS), bicalutamide was discontinued, and the serum PSA level decreased again to less than 0.1 ng/mL. The patient is currently undergoing LH-RH agonist monotherapy and his serum PSA level has been maintained at less than 0.1 ng/mL for more than 30 months without any clinical symptoms (Fig. 1c).

## Conclusion

We present the case of a patient in whom a clinical flare caused by an LH-RH agonist was not prevented by prior anti-androgen administration. In addition, this case demonstrated a prolonged progression free survival (PFS) period (more than 30 months and continuing) of anti-androgen withdrawal syndrome. Interestingly, the serum PSA nadir (0.1 ng/mL) during LH-RH agonist monotherapy was 10-times less than the serum PSA level (1.0 ng/mL) during concomitant anti-androgen and LH-RH agonist administration.

Although LH-RH antagonists are available, the most common form of hormonal treatment is LH-RH agonist therapy. However, during the initial 1–2 weeks, a biochemical and occasionally a clinical flare can occur [1–3]. To prevent the flare-up phenomenon, it is generally thought that prior anti-androgen administration can prevent the biochemical and clinical flare [1–3]. However, in the CS21 phase III Degarelix clinical trial, a smaller PSA surge was noted in the leuprolide patients, even those who were receiving concomitant anti-androgens [4]. In this trial, the authors cautioned that addition of an anti-androgen to a LH-RH antagonist does not always prevent the flare [4]. Oh et al. recently reported that the rates of clinical flare, including fractures, spinal cord compression, bladder outlet obstruction and narcotic prescriptions, were rare in the first 30 days after beginning LH-RH agonist therapy regardless of whether or not anti-androgens are used [5]. In this study, they described their question as: “Does oral anti-androgen use before LH-RH agonists in patients with metastatic prostate cancer prevent clinical consequences of a testosterone flare?” [5]. Therefore, we present a valuable case here.

Anti-androgen withdrawal syndrome is also important to discuss. Anti-androgen withdrawal syndrome was described for the first time by Scher and Kelly [6]. It is known that approximately one-third of patients respond to anti-androgen withdrawal for a median duration of 4 months [1, 6, 7]. In the SWOG 9426 trial, it was reported that the median PFS was 3 months [7]. In addition, 19% patients had a PFS of 12 months or greater, although all of these patients did not have metastatic disease (M0) [7]. In this study, factors associated with increased PFS and overall survival (OS) were longer period of anti-androgen use (more than 10 months), lower PSA at baseline (less than 10 ng/mL) and non-metastatic disease [7]. In our case, the patient’s initial PSA is 974.8 ng/ml, the period of anti-androgen use was 12 months and the patient had multiple bone metastases. Only one of these factors applied somewhat to our patient. However, in our patient, the withdrawal state continued for more than 30 months. In addition, the PSA serum nadir level during the concomitant anti-androgen administration is 10-times more than the level observed during LH-RH agonist monotherapy. Although no study has compared the PSA level nadir during the concomitant administration of anti-androgens and during LH-RH agonist monotherapy, our case shows a prolonged period of AWS and failure to prevent the flare-up. This suggests that, in this case, bicalutamide was ineffective from the first administration.

In conclusion, we reported the case of a patient whose clinical flare could not be prevented or reduced by prior use of the anti-androgen, bicalutamide. With this valuable experience, we re-confirmed that an LH-RH antagonist and surgical castration is a more reliable clinical approach for the prostate cancer patients with symptomatic metastatic disease.

## Consent

Written informed consent was obtained from the patient of this Case report and any accompanying images. A copy of the written consent is available for review by the Editor of this journal.

## Abbreviations

LH-RH: luteinizing hormone-releasing hormone
PSA: prostate specific antigen
AWS: anti-androgen withdrawal syndrome
EOD: extent of disease
PFS: progression free survival
